# Two Different *PRKN* Compound Heterozygous Variants Combinations in the Same Family

**DOI:** 10.1002/mdc3.13725

**Published:** 2023-03-20

**Authors:** Margaux Biehler, Jean‐Marie Ravel, Mélissa Tir, Nadège Calmels, Audrey Schalk

**Affiliations:** ^1^ Laboratories of Genetic Diagnosis Institut de Génétique Médicale d'Alsace (IGMA), Strasbourg University Hospitals Strasbourg France; ^2^ Department of Neurology Amiens University Hospital Amiens France

Bi‐allelic *PRKN* variants are involved in 34% to 45% of familial recessive early‐onset Parkinson's diseases,^1^, ^2^ also called PARK‐Parkin (MIM #600116).^3^ PARK‐Parkin differs from idiopathic Parkinson's disease (PD) in the age onset before 45 years, dystonia at presentation, less frequent dementia, slower progression, better levodopa‐responsivity, and a limited dopaminergic neuron depletion.^2^, ^3^, ^4^

A vast mutational spectrum in *PRKN* has already been noticed, including all types of CNV (copy number variant) and SNV (single nucleotide variant).^5^ Here, we report four affected members of a family carrying two combinations of bi‐allelic *PRKN* pathogenic variants.

The proband (II.2) is the second child from non‐consanguineous parents (Fig. 1A). At 40, he developed typical slowly progressive levodopa‐responsive parkinsonism starting with right hand tremors. The disease have been stable for 20 years, and persists with asymmetric predominantly right‐sided akineto‐rigid syndrome (ARS) with tremors. At 69, he displayed body bradykinesia, moderate segmental akinesia and minimal impairment of postural stability without cognitive, psychobehavioral, or impulse control impairment. His sister (II.4) also displayed typical levodopa‐responsive parkinsonism from age 42 with right foot akinesia and significant freezing of gait. His brother (II.1) displayed typical parkinsonism with chronic psychosis. Disease onset was difficult to precise because of previous neuroleptic‐induced parkinsonism. Observation of asymmetrical parkinsonism around age 55 suggested instead degenerative parkinsonism, which was then confirmed by a severe dopaminergic depletion in the Datscan. At the age of 71, he displayed severe parkinsonism with tremors without postural instability. The proband's daughter (III.1) developed left foot akinesia and left arm tremors at 30 years old, approximately 10 years before other family members, and Datscan showed bilateral putaminal dopaminergic depletion (Fig. 1B). At 37, she displayed a persistent bilateral predominantly left‐sided ARS with typical rest tremors and dystonic posture with clawed toes. Her disease progressed slowly without significant axial signs nor cognitive status or impulse control disorder.

**FIGURE 1:**
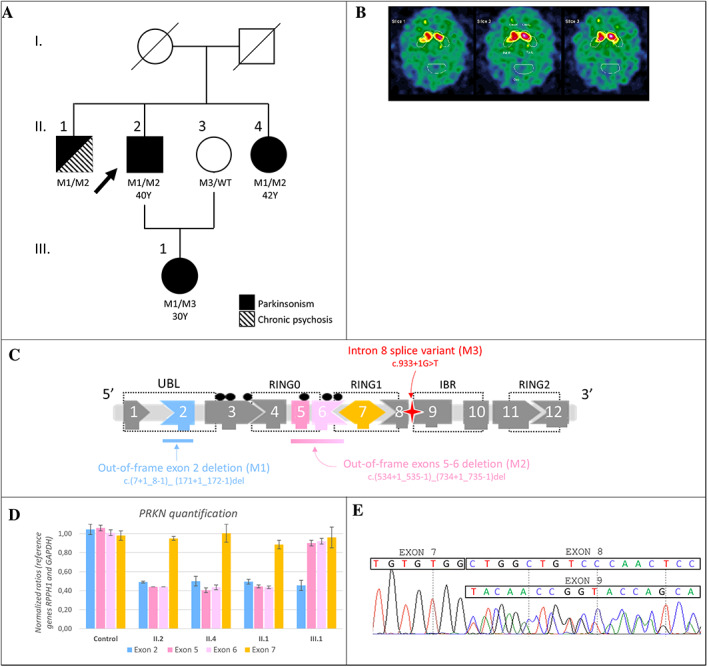
(**A**) Family pedigree. Black symbols represent affected individuals. The arrow indicates the proband. Mutational status: M1: deletion in exon 2 (NM_004562.2:c.(7+1_8–1)_(171+1_172–1)del), M2: deletion overlapping exons 5 and 6 (NM_004562.2:c.(534+1_535–1)_(734+1_735–1)del), M3: splice variant in intron 8 (NM_004562.2:c.933+1G>T), WT: wild type, Y: age at disease's onset. (**B**) Datscan imaging of patient III.1 shows bilateral putaminal dopaminergic depletion. (**C**) *PRKN* structure and variants identified in the family. UBL: ubiquitin‐like domain; RING: zinc finger domain; IBR: in‐between ring domain; black dots: casein phosphorylation sites. (**D**) *PRKN* quantification by qPCR in II.1, II.2, II.4, III.1 and a healthy individual as control. (**E**) Sanger sequencing on III.1 blood cDNA illustrates heterozygous exon 8 skipping. At the end of exon 7, exon 8 and 9 sequences overlap.

Screening of 127 genes involved in movement disorders^6^ revealed two heterozygous intragenic *PRKN* deletions in the proband II.2 (referred to PMD283 in^6^): NM_004562.2:c.(7+1_8–1)_(171+1_172–1)del in exon 2 and c.(534+1_535–1)_(734+1_735–1)del overlapping exons 5 and 6. These two deletions are out‐of‐frame^5^, ^7^ (Fig. 1C). Quantitative PCR (qPCR) confirmed both deletions in II.2, identified them in II.1 and II.4 (Fig. 1D) but retrieved only exon 2 deletion in III.1. Next‐Generation Sequencing of III.1 DNA identified an additional heterozygous substitution in *PRKN* intron 8 (NM_004562.2:c.933+1G>T) inherited from her healthy mother (II.3). Localized on a canonic donor splicing site, this variant induced an out‐of‐frame exon 8 skipping as revealed by RT‐PCR performed on blood cDNA (Fig. 1E). Finally, proband II.2 and siblings II.1 and II.4 carry two different *PRKN* deletions in *trans*, whereas III.1 is compound heterozygous for paternal exon 2 deletion and maternal splicing variant.

We report four relatives displaying PARK‐Parkin related to two combinations of *PRKN* pathogenic variants.

To our knowledge, our report is the first to describe two sets of *PRKN* pathogenic variants CNV/CNV and CNV/SNV in the same family. This report highlights that different molecular mechanisms can induce one disease, even in the same family. It encourages geneticists to consider a recessive pathology even when dominant inheritance is suggested and supports the importance of combining CNV and SNV analysis.

The hypothesis that heterozygous *PRKN* variants are risk factors of classical PD was definitively ruled out recently.^8^ Then our report strongly supports the re‐assessment strategy of possible additional gene mutations in patients with single *PRKN* variant and familial PD.

Bi‐allelic *PRKN* variants are involved in PARK‐Parkin and a vast mutational spectrum has already been described. However, no correlation between the severity and/or precocity of clinical involvement and the nature of the variants in *PRKN* has been established. Nevertheless, incomplete penetrance or variable expression has already been reported,^9^, ^10^ for example, in a family with five *PRKN* compound heterozygous relatives including one asymptomatic member.^9^ Phenotype could also be modulated by variants in other key genes belonging to the parkin pathway.^9^, ^10^ Such compensatory mechanism has already been suggested considering an additional *PINK1* variant enhancing *PRKN* mutations.^3^

The daughter developed PARK‐Parkin around 10 years before other relatives in the present family. Although *PINK1* was included in the targeted genes panel with no anomaly detected, all genes among the parkin pathway have not been explored, and additional variant in a non‐tested gene cannot be ruled out. This difference in age at onset could also be explained by the C‐terminal position of the splice variant inducing abnormal protein production, compared to the absence of protein induced by the N‐terminal deletions.^4^ For example, the most severe phenotype in Erer's cohort was reported with heterozygous exon 11 nonsense variant associated with a non‐pathogenic variant in intron 4.^1^ Protein expression assays such as Western Blot could be interesting to support this hypothesis. In any case, other similar cases are requested to confirm this hypothesis.

## Author Roles

(1) Research project: A. Conception, B. Organization, C. Execution; (2) Clinical Management; (3) Manuscript: A. Writing of the first draft, B. Review and Critique.

M.B.: 1C, 3A

J.M.R.: 1C, 3A

M.T.: 2

N.C.: 1A, 1B, 3B

A.S.: 1A, 1B, 3B

## Disclosures

**Ethical Compliance Statement:** The authors confirm that ethical approval from an institutional review board was not required. Informed patient consent was obtained for this work. We confirm that we have read the Journal's position on issues involved in ethical publication and affirm that this work is consistent with these guidelines.

**Funding Sources and Conflicts of Interest:** No specific funding was received for this work. The authors declare that there are no conflicts of interest relevant to this work.

**Financial Disclosures for the Previous 12 Months:** The authors declare that there are no additional disclosures to report.
